# User-Centered Design and Evaluation of a “Lehuo Pingpang” Exergame for Older Adults With Mild Cognitive Impairment: Cross-Sectional Convergent Mixed Methods Study

**DOI:** 10.2196/93081

**Published:** 2026-08-14

**Authors:** Xi Chen, Hongting Ning, Lin Peng, Chi Zhang, Jiaji Zhang, Dian Jiang, Xiaoyang Li, Xiao Wan, Jundan Huang, Qi Xie, Weiping Huang, Juanfang Jiang, Hui Feng

**Affiliations:** 1 Xiangya School of Nursing, Central South University, No. 172, Tongzipo Road, Yuelu District, Changsha, 410013, China, 86 15173121969; 2 School of Nursing, Hunan University of Chinese Medicine, Changsha, China; 3 School of Food and Chemical Engineering, Shaoyang University, Shaoyang, China; 4 Ningbo Institute of Materials Technology and Engineering, Chinese Academy of Sciences, Ningbo, China; 5 Teaching and Research Section of Clinical Nursing, Xiangya Hospital, Central South University, Changsha, China; 6 Osteoarthropathy Rehabilitation Center, Xiangya Bo’ai Rehabilitation Hospital, Changsha, China

**Keywords:** mild cognitive impairment, older adults, exergames, physical activity, user-centered design, usability

## Abstract

**Background:**

Physical activity is one of the preferred interventions for mild cognitive impairment (MCI); however, the physical activity levels and adherence of older adults with MCI are particularly limited. Exergames are expected to be a key strategy to address these issues. However, existing exergames lack usability among the older adult population.

**Objective:**

We aimed to design and develop an MCI-centered exergame for older adults and test its usability.

**Methods:**

We used a cross-sectional, convergent mixed methods study with a single-group observational design, comprising a user-centered design phase (part 1) and a usability evaluation phase (part 2). In part 1, the “Lehuo Pingpang” virtual reality (VR) table tennis exergame prototype was designed and developed using Unity3D (Unity Technologies) and a PICO 4 Pro system. In part 2, a total of 12 older adults (aged ≥60 years) with MCI were recruited via convenience sampling from community, home-based, and institutional care settings in Changsha, Hunan. Usability was evaluated using a convergent mixed methods approach. Immediately after a supervised, independent gaming session, quantitative usability was measured using the System Usability Scale, while qualitative feedback regarding user experiences and barriers was gathered concurrently through face-to-face semistructured interviews. Data integration occurred at the interpretive level to synthesize the quantitative metrics with qualitative themes.

**Results:**

In part 1, an original design framework for MCI exergames was constructed. Group discussions selected table tennis as the core activity and PICO 4 Pro as the hardware platform based on clarity and physical comfort. A multidisciplinary expert meeting (n=8) optimized the preliminary plan across six dimensions, resulting in the final prototype Lehuo Pingpang. In part 2, 12 participants (mean age 69.08, SD 6.08 y) evaluated the prototype. The mean System Usability Scale score was 79.38 (SD 12.89; 95% CI 71.19‐87.57), indicating acceptable and reliable usability. Qualitative interviews (data saturation achieved at the 9th interview, with 12 completed) identified 2 main themes and 7 subthemes categorized into positive experiences and deficiencies. Mixed methods integration demonstrated that quantitative usability and learnability indicators strongly converged with qualitative praise for simple operation and safety functions, though complemented by learning friction from controller button complexity and VR unfamiliarity, as well as minor user interface flaws (small buttons). Crucially, while quantitative confidence was high, long-term frequent-use intentions were moderate. Qualitative findings revealed that this was driven by physical discomfort (eg, VR dizziness and facial pressure).

**Conclusions:**

Guided by an original evidence-based design framework, this study successfully developed and evaluated a customized MCI-specific VR exergame. Unlike existing research, this work highlights how multidisciplinary co-design and culturally familiar sport themes can optimize digital rehabilitation. It brings to the field of gerontechnology a replicable development workflow and vital mixed methods insights regarding the paradox between quantitative acceptability and qualitative physical discomfort driven by novelty.

## Introduction

Mild cognitive impairment (MCI) represents a transitional pathological state between cognitive decline associated with normal aging and dementia [1]. It is reported that 42% of the worldwide population aged older than 60 years is affected by MCI [2]. Moreover, the prevalence of MCI increases with advancing age, and its incidence and rate of progression to dementia are projected to continue rising, primarily due to global increases in life expectancy and sedentary lifestyles [3-8]. Therefore, older adults with MCI represent a critical window for preventing and treating dementia, and interventions should prioritize addressing sedentary lifestyle behaviors.

Physical activity is the preferred intervention for MCI, and it is also an effective and economical nonpharmacological approach to reducing the risk of dementia [9,10]. Physical activity can significantly improve cognitive function, physical function, and quality of life in patients with MCI [11,12]. However, the physical activity levels and adherence of older adults with MCI are particularly limited due to disease-related motor and cognitive changes. A systematic review of older adults with MCI and dementia found that the adherence rate to physical activity interventions was as low as 16% [13]. Therefore, while physical activity is the preferred intervention for MCI, older adults with MCI still face problems of insufficient physical activity levels and poor adherence.

Exergaming, an emerging form of physical activity, has the potential to achieve this goal. Exergames are a combination of exercise and games, also known as active video games, which incorporate physical activity into a video game environment through body-controlled movements [14]. Soltani et al [15] described exergaming as a tool to combat sedentary lifestyles. Exergaming can improve exercise adherence and participation by enhancing motivational drive and satisfaction [16], which in turn may exert positive effects on cognitive function improvement [17]. The American College of Sports Medicine has even defined exergaming as the future of physical activity [18]. Thus, as an emerging mode of exercise, exergaming holds promise as a key strategy to enhance physical activity levels and adherence among older adults with MCI.

Despite their potential, existing exergames endure notable limitations. Ongoing reviews of current evidence indicate that many studies rely on outdated and commercially discontinued game systems, such as the Nintendo Wii, Xbox Kinect (Microsoft Corp), or Sony PlayStation [19,20]. Crucially, these commercial systems were never specifically designed for therapeutic or rehabilitation cohorts [19]. Previous studies have shown that existing exergames primarily target young people, and the design of related exergames (eg, Wii and Kinect) is not suitable for older adults. These games often feature colorful and visually busy game interactions, inappropriate music, and highly demanding user interface (UI) navigation [21]. Meanwhile, their design in the physical activity and/or cognitive domains is overly complex and difficult [22,23]. Moreover, most existing exergames are designed for entertainment and recreation rather than based on exercise principles [21]. Therefore, most existing exergames lack usability among older adults. Development should be based on the fundamental principles of exercise and tailored to the specific needs of different target users.

Within the broad field of eHealth system development, user-centered design (UCD) [24] has consistently been the most commonly applied framework [25]. Rooted in the usability requirements of the target population, it holds promise for resolving the identified issues in existing exergames. UCD refers to a series of activities throughout the product development process, including collecting user needs, analyzing user tasks, and conducting iterative rapid prototyping and usability testing to optimize the UI for maximum usability [26]. This approach ensures that products incorporate functions meeting user needs and provide interfaces matching users’ experience and capabilities to support efficient task completion [27]. Usability testing within UCD is a critical precursor to formal intervention, as technological usability is a prerequisite for improving health outcomes [28]. Furthermore, user feedback can be leveraged to identify issues [29], facilitating technical refinement and optimization for better adaptation to target users. Therefore, the UCD process is expected to yield a highly usable product.

In the current methodology, the Multidisciplinary Iterative Design of Exergames (MIDE) framework adopts a multidisciplinary research approach and an iterative method to ensure that each exergame meets the necessary health goals while providing an engaging and easy-to-understand design for its target population [30]. It was proposed by Canadian systems design engineer Yirou Li [30]. It is currently the only exergame design framework that targets older adults and covers the entire lifecycle of exergame design, development, and evaluation, aiming to provide guidelines and considerations related to these processes [30]. This framework includes a three-stage methodology: (1) conducting contextual research to understand the needs of users and therapists through direct interaction and literature reviews; (2) a design and development phase requiring collaboration between therapists and users to cocreate and customize game content; and (3) evaluating the device’s effectiveness, usability, and user acceptance. Guided by this framework, the design of the exergame in this study systematically encompasses these three stages—contextual research (design needs analysis), game design and development, and system evaluation—by using the UCD approach and integrating the feedback of various stakeholders. Consequently, adopting a UCD methodology guided by the MIDE framework to explore user preferences and motivational factors is imperative [21]. As digital technologies evolve rapidly, older adults—particularly those with MCI—face increasingly significant digital barriers. Therefore, there is an urgent need for usability research specifically tailored to this vulnerable population. Therefore, there is an urgent need to conduct usability research on technologies among older populations, particularly in individuals with MCI. Accordingly, guided by the MIDE framework, this study aims to (1) design and develop an exergame centered on older adults with MCI and (2) test its usability among older adults with MCI.

## Methods

### Overview

This study consists of two parts: one is to design and develop an exergame prototype (part 1) based on the research results [31] of the team’s previous analysis of the needs of older adults with MCI for exergames, and the other is to test the usability of the exergame prototype among older adults with MCI (part 2).

### Part 1: Design and Development of an Exergame Centered on Older Adults With MCI

#### Phase 1: Early-Stage Preparations

A multidisciplinary team of 12 researchers was established, comprising experts in geriatric nursing, sports rehabilitation, medical robotics, virtual reality (VR), and human-computer interaction technology, alongside graduate students. Their primary task was to translate design concepts into a tangible product. The target user group was defined as older adults aged ≥60 years with MCI. To accommodate the diverse care landscape in China, the exergames were designed for adaptability across community, institutional, and home-based care settings.

#### Phase 2: Needs Analysis and Group Discussion

First, based on our previous research [31], we clarified the specific requirements of older adults with MCI. These include the need for age-friendly design, scientific validity and safety during exercise, a good gaming experience, simultaneous physical and cognitive training, and the provision of support and training. Based on these identified needs, we proposed an original design framework for exergames targeting older adults with MCI as a primary contribution of this paper. Second, the multidisciplinary team convened for a group discussion to translate this newly developed framework into specific, actionable design strategies. Finally, by integrating the core components of the framework with the outcomes of the discussion, the “Preliminary Design Plan for Exergames Targeting Older Adults With MCI” was formulated.

#### Phase 3: Expert Meeting

While our previous needs analysis (phase 2) extensively involved patients with MCI, they were not directly included in this preliminary design validation phase. The primary reason is that older adults with MCI experience cognitive decline, particularly in domains such as abstract thinking and executive function. Evaluating noninteractive, low-fidelity materials (eg, conceptual PowerPoint [Microsoft Corp] slides or UI wireframes) may pose significant cognitive challenges for them, as it requires a high degree of abstract visualization to conceptualize interactive experiences. Therefore, following established inclusive design practices for cognitively impaired populations, we tailored the traditional UCD process by using a multidisciplinary expert team to bridge this gap. The goal was to rapidly translate abstract requirements into a tangible, high-fidelity prototype that MCI users could actually comprehend and evaluate during the subsequent usability testing.

First, the expert team was selected. To recruit independent, external specialists who were entirely separate from our core research and development team, a purposive sampling method was used. Potential candidates were identified through the authors’ academic networks, affiliated clinical institutions, and professional associations, and were initially contacted via email or telephone to present this study’s objectives and scope. Experts participated voluntarily. The inclusion criteria for experts were (1) those engaged in multidisciplinary research, rehabilitation medicine, sports medicine, geriatrics, geriatric nursing, computer software development, ergonomics, human-computer interaction, VR game development, community medical work, etc; (2) those holding a master’s degree or above; and (3) those with an intermediate professional title or above, or with 10 years or more of specialized work or research experience.

Second, a multidisciplinary expert meeting was convened to evaluate the preliminary design. To ensure quality, comprehensive materials were distributed two weeks in advance to allow for preparation. The session began with a presentation of the research context, objectives, preliminary design plan, and interface designs via PowerPoint, followed by a structured discussion on potential revisions to reach a consensus on the optimization strategies. All feedback and agreed revisions were documented through audio recordings and field notes.

To analyze these collected data, the research team reviewed the audio recordings and field notes postmeeting to systematically extract and categorize the experts’ suggestions into thematic design optimization dimensions. Based on these categorized suggestions, the research team adjusted and modified the preliminary exergame design plan to formulate a revised version. This revised plan was returned to the experts for verification and confirmation to ensure their input was accurately interpreted and implemented. After no further revisions were needed, the final version of the exergame design plan was formed.

#### Phase 4: Development of the Exergame Prototype

##### Development Steps of the Exergame

First, based on the final exergame design plan and combined with development objectives and principles, the interface content, interaction modes, logical processes, etc, of the exergame were clarified. Next, software development was carried out by technical personnel; meanwhile, the first author closely collaborated throughout the process, deeply participated, and completed multiple rounds of operation testing and debugging together with technical personnel, ultimately developing the exergame prototype.

##### Development of the Software End

The development strictly followed the UCD approach, closely aligning with the needs of older adults with MCI to transform the design plan into a physical product. As involving older adults with MCI in testing unstable, raw software builds during the programming phase poses excessive cognitive load and potential safety risks, the first author acted as a surrogate evaluator (user proxy) representing the target users. To implement this approach during the coding process, the technical team directly translated the structured modules, interaction logics, and technical specifications outlined in the final design plan—which were grounded in actual user requirements (phase 2)—into concrete C# code in Unity3D. Throughout this software development phase, rather than using a linear coding process, we used an iterative "code-test-refine" cycle. The first author regularly playtested the evolving builds against the user-centered specifications in the final design plan, identified usability hurdles, and collaborated with technical personnel to make immediate code adjustments before the next milestone.

Code writing and functional implementation were carried out according to the game design plan, with project development based on the PC-oriented Unity3D engine. The development used C# language, used Microsoft Visual Studio as the compilation platform, ran on the Windows 11 (Microsoft Corp) OS, and was supported by hardware including an Intel Core i5 CPU and a high-performance PC workstation.

### Part 2: Usability Evaluation With Targeted Users

#### Mixed Methods Research Design Overview

**Figure 1.**
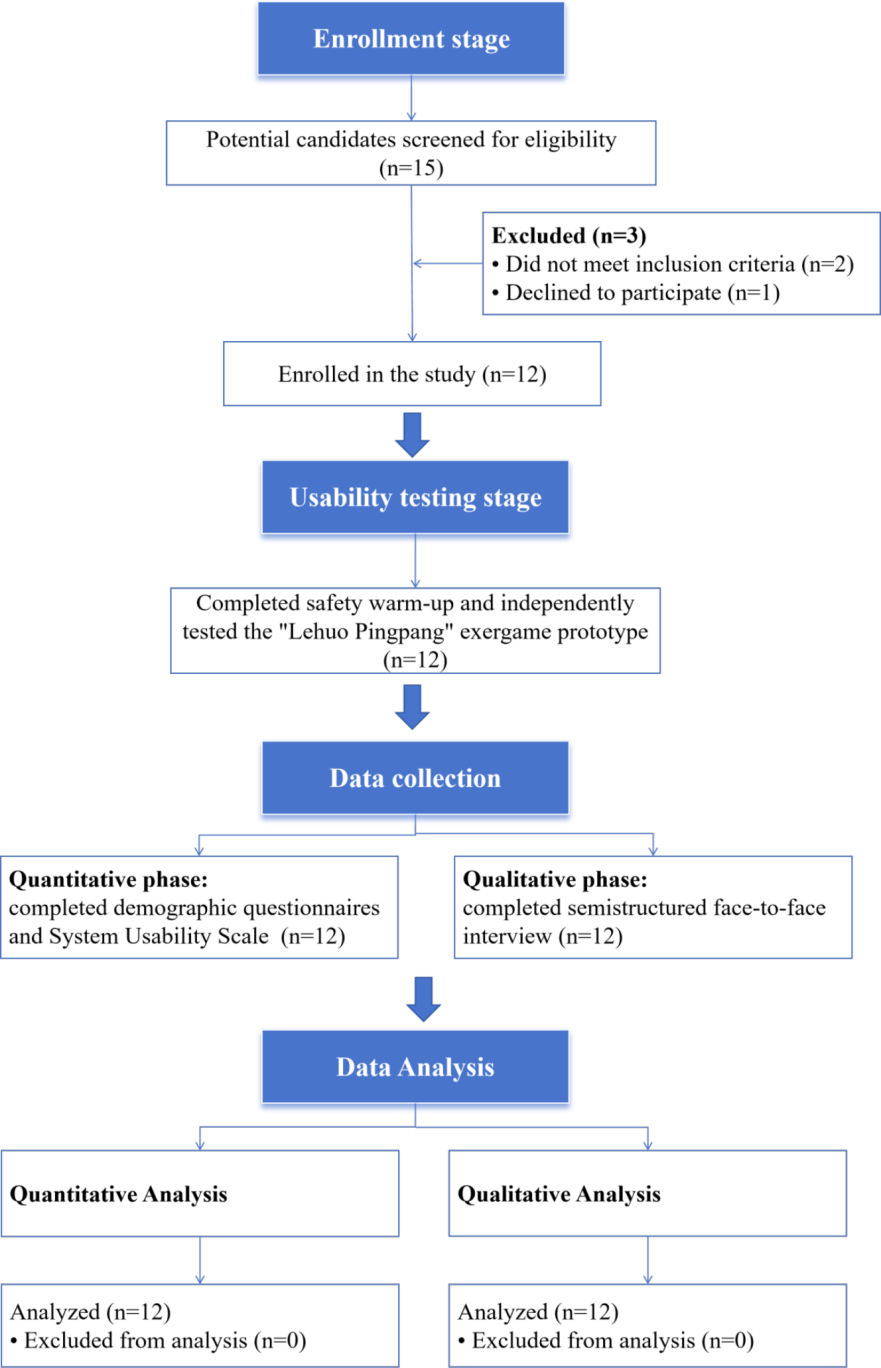
Participant flowchart illustrating the enrollment, usability testing, mixed methods data collection, and data analysis stages for older adults with mild cognitive impairment.

Tuena et al [32] proposed that in usability studies targeting older adults, the mixed research method combining quantitative and qualitative research is the preferred approach, as it can ensure the completeness and interpretability of research results. Thus, to achieve a comprehensive usability evaluation, a cross-sectional, convergent mixed methods design (incorporating a nonexperimental, single-group observational quantitative strand and a descriptive qualitative strand) was used. This study has been reported in line with the GRAMMS (Good Reporting of a Mixed Methods Study) checklist [33]. In this design, quantitative and qualitative data were collected concurrently, analyzed separately, and integrated during the interpretation phase [34]. There were no missing data in this study; all 12 participants completed the required assessments. The flow of participants through each stage of the current study is visually detailed in the participant flowchart (Figure 1).

#### Quantitative Method

##### Inclusion and Exclusion Criteria

Inclusion criteria: aged ≥60 years; met Petersen’s MCI criteria [35], including self-reported or informant-reported memory decline, Montreal Cognitive Assessment (MoCA) scores [36] (≤13 points for illiterate individuals, ≤19 points for those with primary school education, and ≤24 points for those with junior high school education or above), Activities of Daily Living Scale score ≤23, and no clinical diagnosis of dementia. Exclusion criteria were cardiovascular diseases, visual impairments, orthopedic or neurological diseases affecting participation in exergames; cancer; severe obesity (BMI >40 kg/m²); mental disorders or other conditions severely impairing communication; and participation in other health-related studies. No restrictions were placed on recruitment based on demographic characteristics such as sex or ethnicity.

##### Participant Characteristics

###### Overview

Older adults with MCI were recruited in this study. This study’s sample consisted of 12 older adults with MCI. Their major demographic characteristics (including age, sex, and educational background) and important topic-specific characteristics (MoCA scores) are detailed in the Results section.

###### Sampling Procedures

A convenience sampling method was used to recruit participants from community, home, and institutional care settings in Changsha, Hunan, China, between February and March 2024. To identify potential participants, the research team actively collaborated with community health care workers and facility administrators to conduct an initial records-based prescreening of registries. Eligible older adults who met the baseline age and diagnostic records were contacted via telephone or face-to-face visits. The researchers clearly explained the study’s background, objectives, and voluntary participation nature. For those who expressed interest, a face-to-face evaluation session was conducted by the first author in a quiet room to perform the MoCA screening and verify study eligibility. Written informed consent was formally obtained from each eligible individual before enrollment, and they were informed of their right to withdraw at any stage of the usability testing without any negative consequences. No monetary payments or agreements were made to the participants. Out of all eligible individuals who met the prescreening criteria and completed the initial evaluation, 100% participated in the usability testing session.

### Sample Size, Power, and Precision

Regarding sample size, Macefield [37] suggests a reasonable baseline range for identifying usability issues is 5‐10 participants, while comparative studies aiming for statistically significant results should include 10‐12 participants. Additionally, usability study guidelines indicate that the optimal sample size is 10±2 participants [38]. Thus, to achieve adequate precision and representation, the sample size for the quantitative phase of this study was determined to be 12 participants. No power analysis was performed as no inferential hypothesis testing was planned. No stopping rules or interim analyses were used.

### Measures and Covariates

The primary outcome measure was usability. Immediately postsession, a self-developed questionnaire collected demographic data, while the System Usability Scale (SUS) [39] assessed the exergame’s usability. The SUS is a scientifically validated, reliable scale (eg, *ω*=0.91 [40]) that is easy to administer [39,41] and frequently used in game research [19,42-45]. It consists of 10 items covering effectiveness (learnability), efficiency (usability), and satisfaction [46], rated on a 5-point Likert scale (1=“strongly disagree,” 5=“strongly agree”) [39,41]. Scores are calculated using a specific algorithm: raw scores for odd-numbered items are subtracted by 1, and for even-numbered items are subtracted from 5. The sum is multiplied by 2.5 to yield a final score (0‐100). Scores ≥70 are considered “acceptable,” with 50‐70 as “marginally acceptable,” and <50 as “unacceptable” [47]. Secondary measures and covariates included demographic data (age, sex, and educational level). All measures collected in this study are included in this report.

### Data Collection

**Figure 2.**
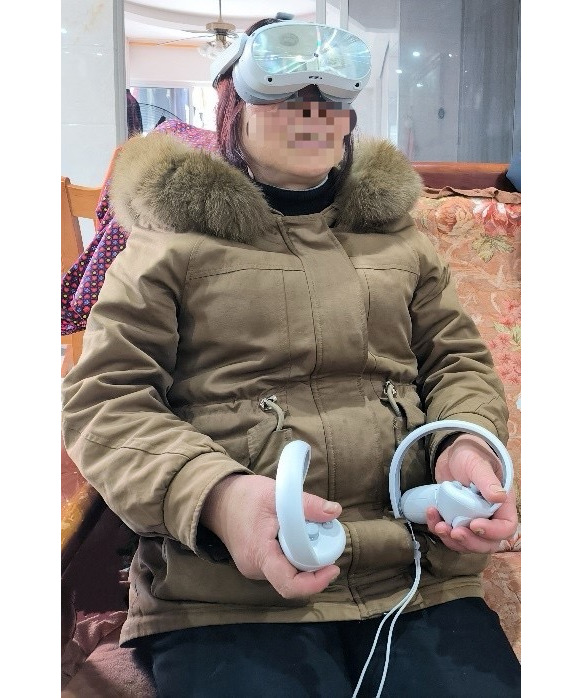
An older adult participant with mild cognitive impairment (MCI) wearing a virtual reality (VR) headset to experience the “Lehuo Pingpang” exergame prototype.

Before this study, the first author (XC) briefed participants on the research objectives and prototype functions, provided a demonstration, and obtained consent for audio recording. Participants selected their preferred testing location (home, community center, or care facility) and performed a mandatory 10-minute safety warm-up. To ensure an authentic experience, participants used the prototype independently under XC’s safety supervision (Figure 2). The quantitative data collection occurred immediately after this session.

### Conditions and Design

This quantitative component of this study used a cross-sectional, nonexperimental, single-group observational design.

### Data Analysis

For quantitative research, valid questionnaires were numbered, and research data were double-entered into Excel (Microsoft Corp) spreadsheets. Quantitative data were analyzed using SPSS (version 26; IBM) software. First, P-P plots were used for normality testing of quantitative data. When quantitative data followed a normal distribution, descriptive statistics were performed using mean and SD; when quantitative data did not follow a normal distribution, median, IQR, minimum, and maximum values were used for descriptive statistics. Frequencies and constituent ratios were used to describe categorical data.

### Qualitative Method

#### Research Design Overview (Qualitative)

The qualitative strand of this study used a descriptive qualitative approach using face-to-face semistructured interviews. This design was chosen to capture participants’ subjective usability experiences, emotional responses, and specific suggestions for prototype refinement. The reporting of qualitative data adhered to the COREQ (Consolidated Criteria for Reporting Qualitative Research) checklist [48].

#### Study Participants or Data Sources

The qualitative phase included the same 12 older adults with MCI who completed the quantitative usability phase, ensuring complete integration of the datasets. The interviews were conducted immediately following the quantitative data collection between February and March 2024.

#### Participant Recruitment

Participants were recruited concurrently with the quantitative phase. After completing the physical exergame play session and the quantitative questionnaires, participants were immediately invited by the researchers to participate in a follow-up interview. Written informed consent for both the interview and audio recording was confirmed once more before the start of the qualitative session.

#### Data Collection (Qualitative)

Face-to-face semistructured interviews (lasting 30‐45 min) were conducted by trained researchers (XC and HN) in a private room at the participants’ testing locations to explore experiences and identify usability issues. The interview guide, developed based on our research objectives, included three open-ended questions: (1) What was your overall experience and how did you feel after using the exergame prototype? (2) Which aspects of the exergame prototype require improvement or optimization? (3) Do you have any additional feedback or suggestions? Audio recordings were transcribed verbatim within 24 hours by the same researchers, supplemented by field notes for analysis.

#### Qualitative Data Analysis

Qualitative data were managed via NVivo (version 12; Lumivero) and analyzed concurrently with collection using thematic analysis [49], which is a highly appropriate method for identifying usability patterns and user experiences. To capture the participants’ experiences directly, a purely inductive coding approach was used, allowing codes and themes to emerge directly from the raw transcript data. This systematic 6-phase process [50] included the following: (1) regarding familiarization, two researchers (XC and HN) independently read the transcripts; (2) regarding generating initial codes and the codebook, the two researchers (XC and HN) independently coded a subset of the transcripts, and then met to compare their open codes, resolve discrepancies through discussion, and establish a preliminary coding scheme (initial codebook); (3) regarding searching for themes, the remaining transcripts were coded in NVivo (version 12) using this preliminary codebook, and related codes were clustered to identify broader candidate subthemes and themes; and (4) reviewing themes, (5) defining and naming themes, and (6) producing the report. Disputed areas in coding or theme interpretation were discussed by the two research team members and resolved through consensus, with consultation from a qualitative research expert (HF).

#### Methodological Integrity

The principal investigator and interviewer (XC) is a doctoral researcher in geriatric nursing and digital health. She has received systematic training in qualitative methodologies and completed a six-month collaborative research residency within the Sports Rehabilitation and Medical Robotics team at the Ningbo Institute of Materials Technology and Engineering. This interdisciplinary background spanning clinical care and medical robotics significantly enhanced the depth of data collection and enriched the interpretation of human-computer interaction nuances during the interviews. The coauthors (HN and HF) are established experts in geriatric nursing, digital health, qualitative methods, and nursing informatics. This collective expertise served to enhance the rigor of qualitative interpretation. Throughout the research process, researchers XC and HN maintained reflexive journals to critically monitor personal assumptions, and the team held regular reflexivity meetings to ensure that all analytical outputs remained strictly grounded in the raw data.

To ensure deep allegiance to the authentic experiences of older adults with MCI, several measures were taken. First, the interviews were conducted immediately following the exergame playing session, ensuring that participants’ recall of usability friction and emotional experiences was fresh and vivid. Second, all interviews were conducted face-to-face in the ecological environments where participants had just completed the gameplay, minimizing cognitive displacement. Finally, raw verbatim transcripts and extensive field notes were continuously cross-checked to ensure that the developed codes fully captured the nuances of the participants’ voices.

To ensure that the qualitative findings directly served our research goal of optimizing the exergame prototype, we focused on actionable utility. The inductive codes were systematically synthesized into distinct optimization categories, which directly mapped onto the design modules for software revision. Triangulation with a qualitative research expert (HF) was conducted to verify that the final themes and subthemes provided concrete, practical design recommendations rather than generic descriptions.

The trustworthiness of this study was evaluated according to four criteria by Lincoln and Guba [51]: (1) credibility, (2) dependability, (3) confirmability, and (4) transferability. A variety of methods were used to ensure trustworthiness. These included taking reflective notes, triangulating data from multiple perspectives (researcher triangulation), conducting research team meetings, reporting on this study in detail, and using verbatim quotes to support themes. In addition, the final themes and results were sent to participants for validation and approval.

### Mixed Methods Integration

Integration occurred at the interpretation level to identify convergence, divergence, and complementarity between the two datasets. Specifically, a joint display was used to systematically compare the quantitative SUS dimension scores with the corresponding qualitative themes and verbatim quotes. While the quantitative SUS scores established a baseline measurement of general usability, the qualitative findings were integrated to explicitly explain the underlying reasons (the “why”) behind these scores and the specific user pain points, thereby generating comprehensive meta-inferences that extend the understanding of the statistical patterns [52].

### Ethical Considerations

This study was approved by the ethics review committee of Nursing and Behavioral Medicine Research, School of Nursing, Central South University (E202397). All participants provided written informed consent before enrollment, acknowledging their right to withdraw at any time without explanation, and were advised that such withdrawal would not affect future services or opportunities. To ensure participant privacy, all collected data were systematically deidentified. Specifically, participants were assigned unique alphanumeric codes upon enrollment, which were used to label all demographic questionnaires, usability scales, and qualitative transcripts. During the verbatim transcription of the qualitative interviews, any potential identifying information mentioned by the participants was removed and replaced with generalized placeholders. Furthermore, all digital files were stored on a password-protected and encrypted storage system accessible exclusively to the core research team. No compensation was offered to the participants. Furthermore, no individual participants or users can be identified in any images included in the manuscript or supplementary material.

## Results

### The Findings of Phase 2 in Part 1

While our prior qualitative research [31] identified the specific exergame requirements of older adults with MCI, the current manuscript proposes an original, structured design framework based on those requirements. As a primary contribution of this paper, this original framework was constructed to guide the subsequent development process. See details in Table 1.

**Table 1.** Design framework for exergames tailored to older adults with mild cognitive impairment.

Theme and subtheme	Module	Category
Design older people-friendly exergames		
Appropriate game rhythm and speed	Adaptive difficulty adjustment	N/A^a^
Simple and easy-to-understand game operations	Error tolerance design	N/A
Simple and easy-to-understand game operations	Game interface design	Color, font, layout, aesthetics, operation
Can be played both sitting and standing	Movement posture	Standing and sitting postures
Ensure scientific validity and safety in the process of sports		
Specify precautions	Safety design	Target users Exercise precautions
Exercise intensity and duration reminder	Safety design	Exercise intensity indication
Exercise intensity and duration reminder	Exercise prescription	Exercise frequency Exercise duration Exercise duration Exercise progression
Provide a good gaming experience		
Link to real life	Game scene	Location, character, costume, prop, ground, background music, sound effects, eye protection mode
Diversified choices	Randomness	N/A
Diversified choices	Game scene	Location, character, costume, prop, ground, background music, sound effects, eye protection mode
Exercise physical and cognitive functions		
Achieve personalization	Adaptive difficulty adjustment	N/A
Exercise physical function	Before and after game assessment	N/A
Exercise cognitive function	Data monitoring	N/A
Exercise cognitive function	Training goals	Daily goals, weekly goals
Exercise cognitive function	Exercise data	Exercise report exercise records
Provide support and training		
Providing feedback and motivation in the game	Feedback mechanism	N/A
Providing feedback and motivation in the game	Reward mechanism	Text and voice encouragement, game score, reward stars, badges
Game usage training	Game modes	Single/double player mode One-handed/two-handed mode Training/competition mode

a N/A: not applicable.

The group discussion established the goal of developing a user-centered, immersive, and culturally relevant exergame. Table tennis was selected as the primary sport activity for three reasons: (1) its familiarity among Chinese older adults minimizes the learning curve and cognitive load; (2) its competitive nature effectively motivates physical engagement [53,54]; and (3) racket sports are the sports most conducive to health and longevity [55]. Furthermore, to accommodate the cognitive limitations of older adults with MCI, the design prioritizes simplified tasks over complex narratives to minimize frustration and ensure training continuity.

**Figure 3.**
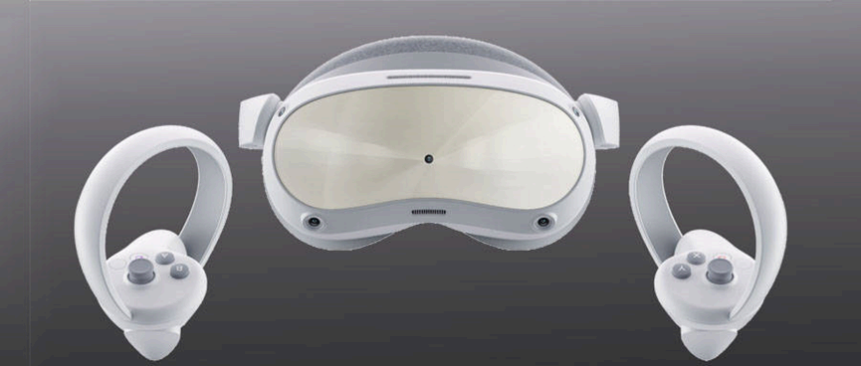
Front view of PICO 4 Pro [56].

Finally, the group discussion selected the PICO 4 Pro (Figure 3) based on two critical metrics: clarity and comfort. Regarding clarity, the device offers 4K+ resolution and a high refresh rate, meeting the requirements for high-quality software streaming. Regarding comfort, it excels in three aspects: (1) supporting interpupillary distance adjustment and allowing users to wear prescription glasses; (2) combining a lightweight body (597 g) with a rear-mounted battery design to achieve front-rear balance; and (3) using a breathable face mask to prevent excessive perspiration during exercise.

Based on the above results, the “Preliminary Design Plan for Exergames Targeting Older Adults With MCI” has been formulated, as detailed in Table 2.

**Table 2.** Preliminary Design Plan for Exergames Targeting Older Adults with Mild Cognitive Impairment. This game uses virtual reality equipment and interacts with the exergame via controllers, eliminating the need for gesture-based interaction. As such, it does not involve the "use of appropriate gestures" principle in exergame development. Concerning the "avoidance of small objects" principle, compared to other table tennis exergames, this game has appropriately increased the size of the table tennis ball and reduced its speed. Different ball sizes and speeds are also set according to varying game difficulties and stages.

Module and category	Content
Game name	
N/A^a^	Lehuo Pingpang
Game rules	
N/A	11-point system
Game modes	
Single/double player mode	Play alone or with another person
One-hand/two-hand mode	Allows using either hand to play the exergame
Training/competition mode	Training mode for learning, competition mode for matches
Pre- and postgame assessment	
N/A	Physical and cognitive functions are assessed through a smart health management system for older adults developed by the research team, which shares data with the exergame
Exercise prescription	
Exercise frequency	At least 2 exercise sessions per week
Exercise time	25 to 60 minutes of daily exercise, with a maximum daily exercise duration of 1 hour
Exercise intensity	At least moderate-intensity exercise, reaching at least 60% of the maximum heart rate
Exercise progression	First increase daily exercise time, then exercise frequency
Exercise postures	
Standing/sitting posture	Use either standing or sitting posture to play the exergame, and switch between them at any time
Game scenario	
Venue, characters, costumes, props, floor, background music, sound effects, eye protection mode	Venue: table tennis stadium; original Chinese animated character designs and costumes with consistent style; game props with contrasting colors; floor free of distractions; background music featuring simple rhythms preferred by older adults; sound effects including ball hitting sounds, cheers, and applause; eye protection mode enabled by default
Adaptive difficulty adjustment	
N/A	Adjust difficulty based on game scores. Increase difficulty in the next round for good performance; otherwise, decrease it. Difficulty levels correspond to specific game stages
Data monitoring	
N/A	Heart rate, exercise time, calorie consumption, etc, displayed on the game interface
Feedback mechanism	
N/A	Real-time feedback through text, voice, static images, and dynamic animations
Reward mechanism	
Text and verbal motivation	Positive reinforcement through text and verbal cues
Game scoring	Display game scores on the game interface
Stars and badges	Reward stars, which can be redeemed for corresponding badges when a certain number is accumulated
Safety design	
Suitable users	Specify the range of suitable users
Exercise precautions	Display safety reminders on the interface before starting the game
Exercise intensity prompt	Provide prompts when exercise intensity is excessive
Fault tolerance design	
N/A	No restrictions on hitting height, angle, or position; hitting within a general range is sufficient
Training goals	
Daily and weekly goals	Provide suggestions for daily and weekly goals before starting the game, but completion is not mandatory
Exercise data	
Exercise report	Include scores, exercise time, heart rate, calorie consumption, equivalent to xx steps, etc
Exercise records	Record day xx, session xx of exercise, etc
Game interface design	
Color, font, layout, aesthetics, operation	Clear color contrast between text and images, simple colors for the interface or background; use simplified Chinese with large font, avoid professional terms; layout from left to right and top to bottom, with important information centered; provide realistic graphic visuals and sound effects to create an aesthetic environment through sensory stimulation; simplify operation steps and minimize hierarchical settings
Randomness	
N/A	Include random values for table tennis size and speed

a N/A: not applicable.

### The Findings of Phase 3 in Part 1

#### Basic Information of Experts

A total of 8 experts from relevant disciplines were invited to the expert meeting (Table 3).

**Table 3.** Basic information of experts.

Number	Sex	Educational background	Professional title	Discipline/specialty
1	Female	Doctor	Professor	Geriatric care
2	Male	Master	Chief therapist	Sports medicine, rehabilitation medicine
3	Male	Doctor	Senior engineer	Mechanical engineering, sports rehabilitation, and medical robotics technology
4	Male	Doctor	N/A^a^	Company director specializing in AI and computer vision
5	Female	Doctor	Professor	Clinical medicine expert
6	Female	Master	Chief rehabilitation therapist	Rehabilitation therapy expert
7	Female	Doctor	Professor	Geriatric care
8	Female	Master	N/A	Virtual reality, human-computer interaction

a N/A: not applicable.

#### Content to Be Modified in the “Preliminary Design Plan for Exergames Targeting Older Adults With MCI”

Overall, while the experts affirmed the scientific validity and feasibility of the preliminary design, they proposed specific optimizations across six dimensions. Regarding game rules, they recommended refining the 11-point system to include side-changing service (a standard table tennis rule where players alternate the right to serve every two points) and adding an automatic service function (an accessibility feature where the system serves the ball automatically if the player fails to react within a set timeframe) to accommodate older adults’ slower reactions. For incentives, they suggested incorporating leaderboards to align with standard gaming conventions and boost motivation. In terms of safety, they emphasized the necessity of emergency measures specifically for fall risks. Furthermore, experts advised extending adaptive difficulty adjustments to the two-player mode and enhancing social connectivity features beyond basic multiplayer interaction.

#### Final Design Plan for Exergames Targeting Older Adults With MCI

Based on the above research findings, the “Preliminary Design Plan for Exergames Targeting Older Adults With MCI” was revised by the development team after discussions, while ensuring that its core elements remained unchanged. This process resulted in the “Final Design Plan for Exergames Targeting Older Adults with MCI,” as detailed in Table 4.

**Table 4.** Final design plan for exergames targeting older adults with MCI.

Module and category	Content
Game name	
N/A^a^	Lehuo Pingpang
Game rules	
Competition rules	11-point system
Service rules	A 5-second countdown will start when serving. If the player fails to serve within the time limit, the system will serve automatically
Side-changing service rules	Players shall switch serving sides after serving twice consecutively. When reaching match point, each player shall serve once alternately
Game modes	
Single/double player mode	Play alone or with another person
One-hand/two-hand mode	Allows using either hand to play the exergame
Training/competition mode	Training mode for learning, competition mode for matches
Pre- and postgame assessment	
N/A	Physical and cognitive functions are assessed through a smart health management system for older adults developed by the research team, which shares data with the exergame
Exercise prescription	
Exercise frequency	At least 2 exercise sessions per week
Exercise time	25 to 60 minutes of daily exercise, with a maximum daily exercise duration of 1 hour
Exercise intensity	At least moderate-intensity exercise, reaching at least 60% of the maximum heart rate
Exercise progression	First increase daily exercise time, then exercise frequency
Exercise postures	
Standing/sitting posture	Use either standing or sitting posture to play the exergame, and switch between them at any time
Game scenario	
Venue, characters, costumes, props, floor, background music, sound effects, eye protection mode	Venue: table tennis stadium; original Chinese animated character designs and costumes with consistent style; game props with contrasting colors; floor free of distractions; background music featuring simple rhythms preferred by older adults; sound effects including ball hitting sounds, cheers, and applause; eye protection mode enabled by default
Adaptive difficulty adjustment	
N/A	In single-player mode, the difficulty is adjusted based on game scores: the difficulty of the next round is increased for good performance, and decreased otherwise. Difficulty levels correspond one-to-one with stages. In two-player mode, if the game scores of both sides differ greatly, the size of the table tennis ball for the loser is increased, or the speed of the table tennis ball is slowed down, and the opposite applies to the winner
Data monitoring	
N/A	Heart rate, exercise time, calorie consumption, etc, displayed on the game interface
Feedback mechanism	
N/A	Real-time feedback through text, voice, static images, and dynamic animations
Reward mechanism	
Text and verbal motivation	Positive reinforcement through text and verbal cues
Game scoring	Display game scores on the game interface
Stars and badges	Reward stars, which can be redeemed for corresponding badges when a certain number is accumulated
Leaderboard	After each game round, a leaderboard is displayed
Safety design	
Suitable users	Specify the range of suitable users
Exercise precautions	Display safety reminders on the interface before starting the game
Fall detection	Real-time fall detection: an alarm is triggered when a fall occurs (to ensure clinical reliability and prevent false alarms during intense gameplay, the real-time fall detection algorithm in “Lehuo Pingpang” operates on a 0.1 ms evaluation cycle using the PICO 4 Pro’s tracking data. The software continuously acquires the real-time headset height (ℎ) and calculates the first-order difference between consecutive heights to approximate vertical velocity. A fall alarm is triggered only when two conditions are met simultaneously: (1) the height ℎ remains below 1.3 m (below normal standing height) for three consecutive cycles; and (2) the calculated height difference (velocity) exceeds 0.8 m/s for three consecutive cycles)
Exercise intensity prompt	Provide prompts when exercise intensity is excessive
Fault tolerance design	
N/A	No restrictions on hitting height, angle, or position; hitting within a general range is sufficient
Training goals	
Daily and weekly goals	Provide suggestions for daily and weekly goals before starting the game, but completion is not mandatory
Exercise data	
Exercise report	Include scores, exercise time, heart rate, calorie consumption, equivalent to xx steps, etc
Exercise records	Record day xx, session xx of exercise, etc
Game interface design	
Color, font, layout, aesthetics, operation	Clear color contrast between text and images, simple colors for the interface or background; use simplified Chinese with large font, avoid professional terms; layout from left to right and top to bottom, with important information centered; provide realistic graphic visuals and sound effects to create an aesthetic environment through sensory stimulation; simplify operation steps and minimize hierarchical settings
Social connection	
N/A	Multiplayer games available; chatting, liking, sharing, etc through the online community
Randomness	
N/A	Include random values for table tennis size and speed

a N/A: not applicable.

### The Findings of Phase 4 in Part 1

**Figure 4.**
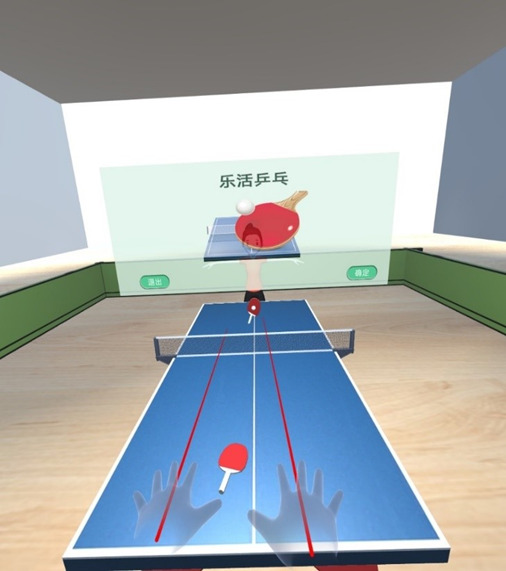
Initial running interface of the Lehuo Pingpang Exergame prototype.

The research and development result is a VR exergame prototype titled Lehuo Pingpang, as shown in Figure 4.

### The Findings of Part 2

#### General Information of Participants

This study included a total of 12 participants, including 7 female and 5 male participants, with a mean age of 69.08 (SD 6.08; 95% CI 65.22‐72.94; range 61‐82) years, as shown in Table 5.

**Table 5.** General information of participants (N=12).

Group	Values
Age (years), mean (SD)	
—^a^	69.08 (6.08)
Sex, n (%)	
Female	7 (58.3）
Male	4 (41.7）
Educational level, n (%)	
Primary school	4 (33.3）
Junior high school	6 (50）
Senior high school	1 (8.3）
Junior college	1 (8.3）
BMI, mean (SD)	
—	23.53 (2.45)
MoCA^b^ scores, mean (SD)	
—	18.75 (2.30)

a Not applicable.

b MoCA: Montreal Cognitive Assessment.

#### SUS Results

The mean SUS score of the Lehuo Pingpang exergame prototype was 79.38 (SD 12.89; 95% CI 71.19‐87.57) points (range 55.00‐97.50, n=12), indicating that the usability of this exergame prototype among older adults with MCI is “acceptable,” that is, it is usable. The narrow margin of this CI, with its lower bound (71.19) remaining comfortably above the established acceptability threshold of 70, indicates a highly precise and reliable conclusion that the exergame prototype has acceptable usability in the target population (Table 6).

**Table 6.** System Usability Scale scores.

Number	Question1	Question2^a^	Question3^b^	Question4^c^	Question5^d^	Question6^e^	Question7^f^	Question8^g^	Question9^h^	Question10^i^	Final score
1	4	1	5	2	5	1	4	1	4	2	87.50
2	3	2	5	2	5	1	5	1	4	4	80.00
3	4	2	5	4	4	2	5	2	5	4	72.50
4	4	2	4	3	5	1	4	1	4	1	75.00
5	3	2	4	4	5	1	4	1	4	4	70.00
6	4	1	5	3	5	1	5	2	5	4	82.50
7	4	1	5	1	5	1	5	1	5	1	97.50
8	5	1	5	1	4	1	5	1	5	2	95.00
9	2	2	3	4	5	1	3	2	3	5	55.00
10	3	2	4	4	5	1	3	2	3	4	62.50
11	5	1	4	2	5	1	4	1	5	2	90.00
12	2	1	5	1	5	1	4	1	4	2	85.00

a Question 2 refers to "2. I find this product too complicated."

b Question 3 refers to "3. I find this product easy to use."

c Question 4 refers to "4. I would probably need support from technical personnel to use this product."

d Question 5 refers to "5. I think the various functions of this product are well integrated."

e Question 6 refers to "6. I think there are too many inconsistencies in this product."

f Question 7 refers to "7. I believe most people could learn to use this product quickly."

g Question 8 refers to "8. I find this product very difficult to use."

h Question 9 refers to "9. I can use this product with ease."

i Question 10 refers to "10. I would need to learn a lot before starting to use this product."

### Results of Semistructured Interviews

#### Overview

**Figure 5.**
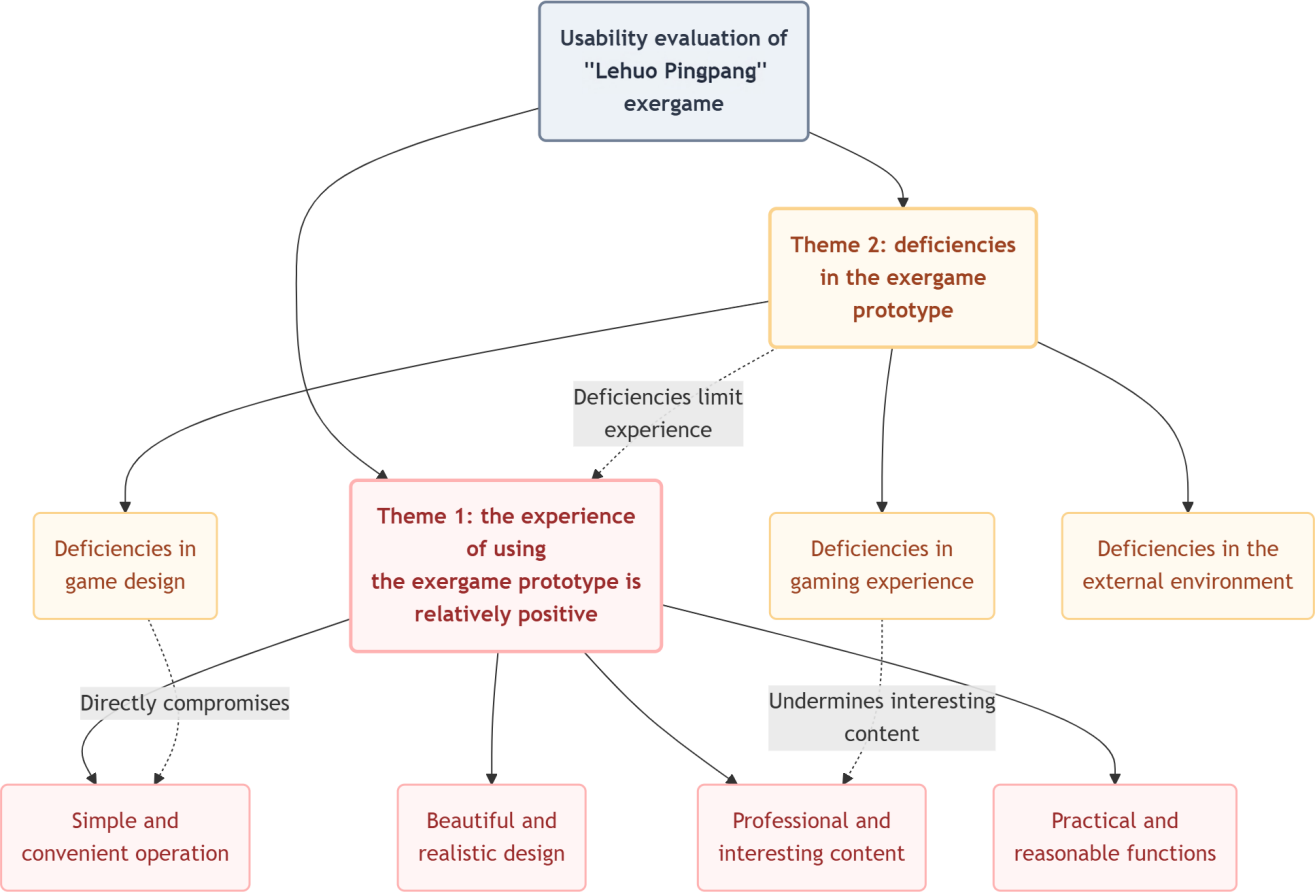
Thematic map illustrating the conceptual network, dynamic interconnections, and analytical relationships between the core usability evaluation focus, the two main themes, and the seven subthemes derived from the reflexive thematic analysis of older adults with mild cognitive impairment.

When the 9th interview was conducted, data saturation was reached. To ensure no new themes emerged, we conducted 3 additional interviews. Therefore, a total of 12 interviews were conducted in this study. Through in-depth analysis of the interview data and induction of categories, themes, and subthemes, a total of 2 themes and 7 subthemes were identified (Figure 5). The older adults with MCI who participated in the interviews were denoted and ordered using the English letter “P.”

#### Theme 1: The Experience of Using the Exergame Prototype Is Relatively Positive

##### Subtheme 1: Simple and Convenient Operation

Some older adults with MCI stated that the game operations, including turning the game on and off, were very simple.

***P2:** Just press the button on the glasses, and you can turn it on or off—just like a phone. That’s really convenient.*

***P8:** The operation is so simple. Anyone who’s played games or used a phone can learn it. It’s even easier than the ones I’ve played before—way simpler, really.*

##### Subtheme 2: Beautiful and Realistic Design

Some older adults with MCI indicated that the colors in the game interface are beautiful and comfortable, and the overall game design is realistic.

***P4:** Wow, everything looks so clear once you put on the glasses. It feels clearer than what I see with my own eyes, just like watching TV or a movie.*

***P5:** This game is exactly like the real thing—totally identical. It’s high-tech stuff.*

***P7:** The colors in this game are really nice. They look comfortable, easy to get used to.*

##### Subtheme 3: Professional and Interesting Content

Some older adults with MCI stated that the game content is professional and consistent with actual rules and norms. In addition, the exergame is highly interesting and can arouse the interest of older adults with MCI.

***P4:** This game is pretty fun, y’know? I really like it. Once I get used to having a good time with it, I’ll keep playing nonstop.*

***P5:** The rules for playing in the game are the same as real table tennis rules—really standard. I’m a pro at this, I know what I’m talking about, haha.*

***P6:** I really like this. I’m quite interested in it.*

***P7:** No matter how many days I play or how long, these goals are basically achievable. They’re all perfect for us older adults.*

##### Subtheme 4: Practical and Reasonable Functions

Some older adults with MCI mentioned that function settings such as the selection of controllers, game feedback, and safety settings in the game are both practical and reasonable.

***P2:** You can use one racket or two—this function is great. Choose whatever you want, either way works.*

***P3:** There’s a reminder if you swing too early or too late. That’s really good. I can learn from it and get better and better.*

***P5:** I love the function that sets a safety zone. Step out of the zone, and you can see real things outside; stay in it, and you only see what’s in the glasses. It makes you feel really secure.*

### Theme 2: Deficiencies in the Exergame Prototype

#### Subtheme 1: Deficiencies in Game Design

Some older adults with MCI stated that the exergame prototype has deficiencies in design, such as font size and clarity.

***P1:** The ‘confirm’ and ‘exit’ buttons need to be bigger. They’re too small—I can never point to them right. Also, they’re way too far apart. After tapping one, I have to turn my head to tap the other. Can’t they be put together?*

***P6:** Everything else is fine, but the characters are a bit blurry. I couldn′t even make out the character′ball′). Its strokes aren′t clear—it looks like it′s blocked by the people in the game.*

***P8:** The space between the ping-pong table and the wall here is too narrow. The people in the game can’t move around freely. It doesn’t make sense.*

#### Subtheme 2: Deficiencies in Gaming Experience

For example, older adults with MCI have poor gaming experience due to their lack of relevant experience, such as using exergames; second, older adults with MCI may experience discomfort when wearing VR headsets; in addition, there are insufficient types of exergames, etc.

***P1:** I’ve never used anything like this before. It just feels awkward to use.*

***P3:** I don’t have other issues, but wearing the glasses for too long makes my head a bit dizzy. Not super dizzy—just that uncomfortable feeling of not being used to it.*

***P7:** It doesn’t feel very comfortable. It’s pinching my face, and that pinch feels unpleasant.*

***P5:** The light inside the glasses is pretty bright. Wearing them for a long time makes my eyes feel strained.*

***P9:** There aren’t enough games here. Add some more, and it’ll be even more fun.*

#### Subtheme 3: Deficiencies in the External Environment

Some older adults with MCI mentioned that the exergame prototype has deficiencies in terms of the external environment, as it is affected by factors such as network and external hardware environment.

***P1:** Is this affected by the network? The Wi-Fi signal at my place isn’t great, and it feels like the stuff in the glasses lags.*

***P2:** I just think there are a bit too many buttons. I can’t remember which one to press when. If there were fewer, it’d be better.*

***P4:** It’s just my feeling—maybe I’m not using it right, but I feel like I’m standing really high up, higher than the ping-pong table. It’s weird. Shouldn’t the game fix this on its own? I should be placed on the floor by the ping-pong table instead.*

### Integration of Quantitative and Qualitative Findings

Following the convergent mixed methods design, the quantitative SUS scores and qualitative themes were integrated using a joint display (Table 7) to generate comprehensive meta-inferences. Based on established usability evaluation frameworks [46], the 10 SUS items were structured into three core dimensions: learnability (items 4, 5, and 10), usability (items 2, 3, 7, and 8), and user satisfaction (items 1, 6, and 9). These quantitative dimensions and their raw item-level scores were mapped directly against the corresponding qualitative themes, subthemes, and illustrative participant quotes to explore convergence and complementarity.

**Table 7.** Joint display of quantitative SUS^a^ scores and qualitative interview themes.

Usability dimension	Mean score (SD; 95% CI)	Corresponding qualitative theme and subtheme	Illustrative quotes	Integration findings (meta-inferences)
1. Learnability	Question 4: 2.50 (SD 1.24; 1.71‐3.29) Question 5: 4.83 (SD 0.39; 4.58‐5.08) Question 10: 2.83 (SD 1.40; 1.94‐3.72)	Theme 1: the experience of using the exergame prototype is relatively positive Subtheme 4: practical and reasonable functions Theme 2: deficiencies in the exergame prototype Subtheme 2: deficiencies in gaming experience Subtheme 3: deficiencies in the external environment	P5 (theme 1): “I love the function that sets a safety zone... It makes you feel really secure.” P1 (theme 2): “I’ve never used anything like this before. It just feels awkward to use.“ P2 (theme 2): “I just think there are a bit too many buttons. I can’t remember which one to press.”	Convergence and complementarity: excellent quantitative functional integration (question 5=4.83) strongly converges with users’ praise for the integrated safety zone function. However, the moderate scores on learning costs and need for support (question 10=2.83, question 4=2.50) are complemented by qualitative findings. Unfamiliarity with VR^b^ hardware (P1) and controller button complexity (P2) created learning friction, explaining the quantitative learning curve.
2. Usability	Question 2: 1.50 (SD 0.52; 1.17‐1.83) Question 3: 4.50 (SD 0.67; 4.07‐4.93) Question 7: 4.25 (SD 0.75; 3.77‐4.73) Question 8: 1.42 (SD 0.51; 1.10‐1.74)	Theme 1: the experience of using the exergame prototype is relatively positive Subtheme 1: simple and convenient operation Theme 2: deficiencies in the exergame prototype Subtheme 1: deficiencies in game design	P2 (theme 1): “Just press the button on the glasses, and you can turn it on or off... really convenient.” P8 (theme 1): “The operation is so simple... Anyone who’s played games can learn it.” P1 (theme 2): “The ‘confirm’ and ‘exit’ buttons need to be bigger. They’re too small.”	Convergence and complementarity: quantitative usability indicators are exceptionally strong, evidenced by high ease of use (question 3=4.50, question 7=4.25) and very low perceived complexity and difficulty (question 2=1.50, question 8=1.42). This strongly converges with qualitative reports of simple, phone-like operations. However, specific UI^c^ design flaws (eg, small “confirm/exit” buttons, P1) complement these scores, highlighting target areas for iterative refinement.
3. User satisfaction	Question 1: 3.58 (SD 1.01; 2.94‐4.22) Question 6: 1.08 (SD 0.29; 0.90‐1.26) Question 9: 4.25 (SD 0.75; 3.77‐4.73)	Theme 1: the experience of using the exergame prototype is relatively positive Subtheme 2: beautiful and realistic design Subtheme 3: professional and interesting content Theme 2: deficiencies in the exergame prototype Subtheme 2: deficiencies in gaming experience	P5 (theme 1): “The rules for playing in the game are the same as real table tennis rules—really standard.” P7 (theme 1): "The colors in this game are really nice. They look comfortable, easy to get used to.” P3 (theme 2): “I don’t have other issues, but wearing the glasses for too long makes my head a bit dizzy. Not super dizzy—just that uncomfortable feeling of not being used to it.”	Discrepancy and expansion: quantitative scores show high confidence in use (question 9=4.25) and low perceived inconsistency (question 6=1.08), converging with praise for standard tennis rules and comfortable visual designs. However, the relatively moderate score on the intention to use frequently (question 1=3.58) contrasts with this high confidence. Qualitative findings reveal that this discrepancy is driven by significant physical barriers (VR dizziness/facial pinching) and is linked to a potential novelty effect and social desirability bias (which initially inflated question 9 and question 6 scores), subsequently exposed and balanced by candid qualitative interviews. This confirms how physical experience limits sustained, long-term satisfaction (question 1=3.58).

a SUS: System Usability Scale.

b VR: virtual reality.

c UI: user interface.

## Discussion

### Principal Findings

This study successfully designed, developed, and evaluated an immersive VR exergame prototype tailored to older adults with MCI. Guided by the three-stage MIDE framework, we successfully developed “Lehuo Pingpang,” translating the target population’s requirements into an aging-appropriate and safe VR exergame prototype. Furthermore, our convergent mixed methods usability evaluation confirmed that the exergame prototype is safe and generally acceptable. By integrating our quantitative SUS scores and qualitative interview themes into three core dimensions—usability, learnability, and user satisfaction—we derived comprehensive insights regarding the prototype’s performance. Collectively, these integrated findings demonstrate that while the exergame prototype is highly viable and acceptable, the identified challenges provide essential, user-driven empirical guidance for subsequent iterative optimizations.

### System Usability: Quantitative Comparison With Prior Work

The average SUS score in this study was higher than the 70-point threshold required for an acceptable game [47], indicating that the usability of the exergame prototype among older adult individuals with MCI is acceptable. These results are consistent with the findings of Altorfer et al [57] and Shah et al [58], who reported even higher average SUS scores. Altorfer et al [57] explored the feasibility and effectiveness of a cognitive-motor dual-task training exergame in 39 older adult inpatients through a pilot randomized controlled trial, testing its usability with the SUS scale. The results showed an average SUS score of 83.60 (SD 13.72), indicating high usability among older adult inpatients. Shah et al [58] co-designed and developed a VR-based exergame with older adults living in or previously living in rehabilitation centers and physical therapists, using a mixed methods approach to evaluate its usability. Among 14 participants, the quantitative part yielded an SUS score of 83.75 (SD 13.3), confirming acceptable usability. The higher SUS scores in these two studies may be attributed to the fact that participants in this study experienced the exergame prototype fewer times and for a shorter duration, whereas the usability studies mentioned above measured SUS scores after multiple long-term exposures, allowing participants to become more familiar with and accepting of the exergame, thus achieving higher usability levels.

However, this study’s results are inconsistent with those of Thalmann et al [59], who conducted a usability study on their newly developed VITAAL exergame among 13 older adults with mobility impairments, reporting an average SUS score of 58.3 (SD 16.5), lower than this study’s findings. Possible reasons include two aspects: on the one hand, the participants in this study had a younger average age, and research by Bangor et al [60] showed a significant correlation between age and SUS scores, meaning older age may negatively impact SUS scores, which could explain why this study’s results were higher. On the other hand, the study by Thalmann et al [59] included older adults with mobility impairments, while the 12 participants in this study had no mobility limitations. Thus, compared to the sample from the study by Thalmann et al [59], this study’s participants may have been more suited to using exergames.

### Multidimensional Mixed Methods Integration: Usability, Learnability, and Satisfaction

In the usability dimension, the low perceived complexity and high ease of use demonstrated by the quantitative analysis strongly converged with the “simple and convenient operation” reported by participants in the qualitative evaluation. Participants generally appreciated the system’s intuitive physical layout and convenient operational experience. This success is primarily attributed to the strict adherence to age-appropriate interface design principles during system development. To accommodate age-related degenerative changes, the prototype incorporated appropriate error tolerance rather than overemphasizing precise manipulation [61]; concurrently, interface elements were arranged in a regular and simplified manner to reduce the attentional burden on older adults [61]; and sufficient reaction time was provided to bolster their confidence [62]. Furthermore, to assist older adults with MCI in processing information rapidly, the interface used large fonts and icons while avoiding complex technical jargon [61]; the layout adhered to visual movement rules of left-to-right and top-to-bottom, presenting crucial prompt information in the center to guide the user’s line of sight [62]; meanwhile, the system integrated multichannel feedback modes, such as audio-visual synchronization and dynamic graphics, to satisfy the information readability needs of older adults [63]. This series of design practices aligns with the design principles of Bogza et al [64] for a web-based decision aid tailored to older adults with MCI, which similarly emphasized simplifying design, using clear and easy-to-understand phrasing, and using pictograms as visual reminders to aid user memory. Moreover, qualitative interviews played a critical complementary role to the aforementioned positive ratings. Under the subtheme of “deficiencies in game design,” some participants pointed out that the “confirm” and “exit” buttons were too small. This complementary finding reveals minor shortcomings in the actual execution of centering and sizing guidelines within the interface layout, thereby providing a precise, user-experience-driven direction for subsequent iterative UI designs.

In the learnability dimension, the prototype received moderate ratings, indicating that some participants faced a distinct learning curve. On one hand, the quantitative high functional integration of the system strongly converged with the qualitative appreciation for the practical safety boundary function, with participants stating that the customizable safety zone provided a profound sense of security. This is scientifically supported by the system’s built-in safety boundary setup: the VR headset first displays the real-world scene through pass-through video, and only presents the virtual world scene after the user has autonomously designated a safe range; when the user exits the safety zone, the device issues a prompt and automatically switches back to the real-world scene, ensuring safety during exercise. Furthermore, to prevent overexertion and injury, sufficient time was allocated between high-intensity exercises for older adults to relax and recover [65]. On the other hand, the higher learning costs and moderate need for technical support shown in the quantitative analysis were critically complemented by qualitative interviews—participants generally mentioned their lack of prior VR experience and expressed confusion regarding the complex controller button layout. The study by Tuena et al [32] pointed out that handheld controllers can be complex for some older adults, particularly those with dementia or MCI; this is consistent with the findings of Malinowski et al [66], who revealed that older adults with MCI face greater difficulties in using technology than those without any cognitive impairments. Additionally, Gerling et al [67] emphasized that providing a technology-based training system capable of building technological confidence is of paramount importance, enabling a better gaming experience and more successful training. Multiple prior studies have similarly confirmed the importance of age-appropriate design and robust technical functionality to the usability of exergames [44,68,69]. Regarding learning costs, Thalmann et al [59] also pointed out that a brief period of familiarization and learning is required during the first training session of an exergame. Collectively, these findings highlight the necessity in future clinical applications of providing structured preuse training, allowing sufficient adaptation time, and designing simplified, tactile controllers customized specifically for older populations.

In the user satisfaction dimension, quantitative indicators exhibited high subjective confidence and extremely low perceived system inconsistency, which strongly converged with the qualitative approval of the prototype’s “beautiful and realistic design” and “professional and interesting content.” A plausible explanation is that table tennis is a highly familiar sport and leisure element in the daily lives of most Chinese older adults, which is crucial for enhancing game acceptance [70] and consequently boosting their confidence to participate. The validation of the prototype’s “beautiful and realistic design” stemmed from the aesthetic sensory stimulation provided by its realistic visual graphics and audio effects [71]; the endorsement of its “professional and interesting content” is consistent with the findings of Shah et al [58], which confirmed through semistructured interviews that older adults find VR-based exergames highly engaging and interesting. The “professionalism” of the system is chiefly reflected in its multidisciplinary, collaborative design philosophy: the development process deeply integrated opinions from various experts across hospitals, universities, research institutes, and technology enterprises, covering four key domains—geriatric nursing, clinical medicine, rehabilitation medicine, and AI; moreover, the entire design scheme was fully evidence-based, strictly adhering to the guiding principles in the “Guidelines for Designing Exergames to Enhance Physical and Social Activity of Older Adults” [61].

However, a prominent methodological discrepancy and contradiction emerged between the exceptionally high subjective confidence and the relatively moderate intention to use the system frequently. Qualitative interviews expanded on this paradox, revealing that participants’ long-term willingness to use the game was likely constrained by immediate physical discomforts such as VR motion sickness (dizziness), eye fatigue, facial pressure, and physical fatigue. These feedback reports of physiological discomfort were validated by the systematic review of clinical and research applications of VR in older adults by Tuena et al [32], which also identified usability issues including discomfort and motion sickness. In addition, research by Stanney et al [72] and Sharples et al [73] pointed out that eye strain is one of the most frequently reported symptoms during the use of VR technology. This concern is further supported by similar exergame research; for instance, in a study by Song et al [74] evaluating the usability and feasibility of VR and mixed reality Tai Chi exergames for community-dwelling older adults, although the physiological comfort of the screened sample was acceptable and severity levels were low, they initially excluded 18.6% of participants due to VR discomfort. Prior studies have emphasized that VR cybersickness is a common but frequently underestimated barrier that can lead to user abandonment [75,76], which explains the underlying reason why participants in our study held reservations regarding long-term, frequent use.

From a deeper perspective, this paradox of high scale scores coexisting with physical discomfort can be explained by the “novelty effect” commonly observed in usability testing with older populations. The enjoyment of the experience stemmed largely from the “novelty” brought by the game [70]. The highly immersive nature of VR was an unprecedented experience for most first-time participants with MCI, and the intense freshness and excitement of the initial trial temporarily masked or diluted their physical discomfort when filling out the quantitative assessments. This key contradiction underscores the indispensable value of the convergent mixed methods design used in this study: the quantitative survey established a positive baseline of general acceptability, while the qualitative interviews successfully unmasked the true physical barriers and technical friction, thereby ensuring a balanced and objective evaluation.

### Iterative Optimization and Future Design Considerations

In response to the usability issues, an iterative design scheme for the exergame prototype is proposed. First, in terms of game design, the “confirm” and “exit” buttons should be enlarged, and the distance between them shortened; text in the game scene should be prevented from being obscured; and the distance between the table tennis table and the gymnasium walls in the virtual scene should be adjusted to a reasonable range.

Studies have shown that compared with nonimmersive devices such as desktop computers and projectors, the use of head-mounted displays is more likely to cause head pressure, eye fatigue, physical fatigue, and cybersickness symptoms [77]. For standalone devices such as the PICO 4 Pro, these symptoms are often influenced by software optimization. The technical specifications of our prototype’s Unity (Unity Technologies) build target a 90 Hz screen refresh rate, a rendering resolution of 1920×1920 per eye, and an end-to-end latency of 12 ms. To address issues such as strong light inside the glasses, eye strain from prolonged use, discomfort from the VR headset feeling tight on the face, and dizziness, future software optimizations will focus on several critical avenues. First, we will implement Application SpaceWarp to significantly reduce rendering latency and improve performance, allowing the application to render at half the actual screen refresh rate without affecting display quality [78]. Second, we will enable Adaptive Resolution to automatically scale the viewport resolution based on the current GPU workload—decreasing it during high GPU loads and increasing it when resources are sufficient—maximizing GPU resources to enhance image quality without dropping frame rates [79]. Third, adopting the Universal Render Pipeline will optimize PICO’s GPU capability and improve rendering performance specifically for XR [80]. While software optimization remains our primary pathway, for users who still experience persistent discomfort, we will consider using a surface television instead of a head-mounted display in future studies to reduce the aforementioned discomfort while maintaining a sense of immersion.

To tackle the problem of limited game types, we will continue to enrich the variety of games in future research to provide more options for older adults. In addition, older adults with MCI require sufficient time to learn, understand, and adapt to the exergame, as well as adequate preuse training, and sufficient support during use. Finally, regarding hardware-related issues of the game, such as a slightly excessive number of controller buttons, susceptibility to network influences during use, and the lack of reminders or guidance for older adults to adjust the relative height between the controller and the ground, continuous improvement and optimization will be made in subsequent processes.

### Methodological and Design Implications for MCI-Centered Exergames

Based on our design and development experience, several key recommendations are proposed for future exergame designs tailored to older adults with MCI. First, ensuring safety during exercise is paramount, which requires adopting a UCD approach throughout the design and development process in close collaboration with stakeholders such as older users and health care professionals. To maximize physical accessibility, the interface must be simple and easy to understand, comfortably accommodating both standing and sitting postures alongside single- or two-handed gameplay. Second, cognitive load should be minimized by incorporating widely familiar elements, providing high error tolerance, and offering sufficient reaction times during play to prevent user frustration. Furthermore, exergames should prioritize health benefits and focus on individual differences, dynamically balancing user abilities and game difficulty through adaptive difficulty adjustments. Finally, to foster long-term engagement, designers should offer diverse task scenarios, use positive incentive strategies while avoiding negative reinforcement, integrate social support functions to enhance social connections, and deliver multichannel feedback with real-time prompts and responses.

### Limitations

Some limitations of this study need to be acknowledged. First, regarding the design and development phase, this study only designed and developed one game scenario and one exergame. Second, there was a lack of direct end user (patients with MCI) participatory co-design during the intermediate preliminary design validation (phase 3). While this tailored UCD approach was considered because the cognitive decline and potential difficulties in abstract thinking in patients with MCI may make it challenging for them to evaluate noninteractive, low-fidelity UI mock-ups, it deviated from a strict UCD cycle. Relying solely on multidisciplinary experts at this stage meant that certain UI issues, such as inappropriate button sizes and spacing, were only identified after the high-fidelity prototype was fully developed. Nevertheless, these identified UI issues served as empirical, user-driven data that directly informed the concrete, multidimensional iterative design scheme proposed in the Iterative Optimization and Future Design Considerations section for subsequent optimization. Future iterations will explore adapted co-design methods with end users earlier in the process, using simplified but interactive mock-ups to prevent such UI failures.

Regarding the usability evaluation phase, several limitations must be noted. First, the sample size of participants was relatively small, and all participants were from the same region, which may lead to selection bias. Additionally, this study only explored the usability of the exergame prototype from the perspective of older adults with MCI, without investigating this issue from the perspectives of other stakeholders (such as caregivers, medical staff, etc). Notably, although the sample size of 12 participants aligns with usability testing guidelines for identifying usability issues, the high SD in the SUS scores (12.89) indicates significant individual differences among older adults with MCI. Future research should involve a larger and more diverse sample size to better account for this variance and to further validate the system’s usability across a broader user group. Finally, the duration of the usability study is relatively short, which may not be able to fully explore the existing usability issues.

### Conclusions

This study successfully designed, developed, and evaluated “Lehuo Pingpang,” a tailored VR table tennis exergame prototype for older adults with MCI. The innovation of this work lies in the formulation of an original, evidence-based design framework that systematically translates the complex cognitive-motor requirements of MCI users into safe, immersive physical-digital interactions. Furthermore, this study contributes to the field of gerontechnology a replicable, multistage development workflow and critical mixed methods methodological insights, unmasking the paradox where high quantitative usability scores can temporarily mask qualitative physical discomfort (eg, VR dizziness and facial pressure) due to novelty effects. In the real world, this prototype provides a highly viable, safe, and motivating dual-task rehabilitation tool. It can be easily deployed across community, institutional, and home-based care settings, ultimately enhancing cognitive-motor capacity, lowering barriers to digital interventions, and promoting active, healthy aging among cognitively vulnerable older populations.

## Supplementary material

10.2196/93081 Checklist 1 GRAMMS checklist.

## Abbreviations

COREQ: Consolidated Criteria for Reporting Qualitative Research
GRAMMS: Good Reporting of a Mixed Methods Study
MCI: mild cognitive impairment
MIDE: Multidisciplinary Iterative Design of Exergames
MoCA: Montreal Cognitive Assessment
SUS: system usability scale
UCD: user-centered design
UI: user interface
VR: virtual reality
