# Supplementary material for: User-Centered Design and Evaluation of a “Lehuo Pingpang” Exergame for Older Adults With Mild Cognitive Impairment: Cross-Sectional Convergent Mixed Methods Study
Source: JMIR Serious Games. 2026 Aug 14;14:e93081. doi: 10.2196/93081 (PMC13477836; doi:10.2196/93081)
Supplement: Supplementary file 1 [file games-v14-e93081-s001.docx]

**Good reporting of a mixed-methods study (GRAMMS) checklist**

| Guideline | Page information |
| --- | --- |
| 1. Describe the justification for using a mixed methods approach to the research question | 7 |
| 2. Describe the design in terms of the purpose, priority and sequence of methods | 7,9 |
| 3. Describe each method in terms of sampling, data collection and analysis | 8-12 |
| 4. Describe where integration has occurred, how it has occurred and who has participated in it | 7,12 |
| 5. Describe any limitation of one method associated with the present of the other method | 11 |
| 6. Describe any insights gained from mixing or integrating methods | 28-32 |

Reference: O'Cathain A, Murphy E, Nicholl J. The quality of mixed methods studies in health services research. J Health Serv Res Policy. 2008;13: 92-98.
